# Procollagen-lysine 2-oxoglutarate 5-dioxygenase 2 promotes hypoxia-induced glioma migration and invasion

**DOI:** 10.18632/oncotarget.15581

**Published:** 2017-02-21

**Authors:** Yangyang Xu, Lin Zhang, Yuzhen Wei, Xin Zhang, Ran Xu, Mingzhi Han, Bing Huang, Anjing Chen, Wenjie Li, Qing Zhang, Gang Li, Jian Wang, Peng Zhao, Xingang Li

**Affiliations:** ^1^ Department of Neurosurgery, Qilu Hospital of Shandong University and Brain Science Research Institute, Shandong University, Jinan 250012, China; ^2^ Institute of Basic Medical Sciences, Qilu Hospital of Shandong University, Jinan 250012, China; ^3^ Department of Neurosurgery, Jining No.1 People's Hospital, Jining 272011, China; ^4^ Department of Biomedicine, University of Bergen, 5009 Bergen, Norway

**Keywords:** hypoxia, procollagen-lysine 2-oxoglutarate 5-dioxygenase 2, FAK, glioma, invasion

## Abstract

Poor prognosis of glioblastoma multiforme is strongly associated with the ability of tumor cells to invade the brain parenchyma, which is believed to be the major factor responsible for glioblastoma recurrence. Therefore, identifying the molecular mechanisms driving invasion may lead to the development of improved therapies for glioblastoma patients. Here, we investigated the role of procollagen-lysine 2-oxoglutarate 5-dioxygenase 2 (PLOD2), an enzyme catalyzing collagen cross-linking, in the biology of glioblastoma invasion. *PLOD2* mRNA was significantly overexpressed in glioblastoma compared to low-grade tumors based on the Oncomine datasets and REMBRANDT database for human gliomas. Kaplan-Meier estimates based on the TCGA dataset demonstrated that high *PLOD2* expression was associated with poor prognosis. *In vitro*, hypoxia upregulated PLOD2 protein in U87 and U251 human glioma cell lines. siRNA knockdown of endogenous HIF-1α or treatment of cells with the HIF-1α inhibitor PX-478 largely abolished the hypoxia-mediated PLOD2 upregulation. Knockdown of *PLOD2* in glioma cell lines led to decreases in migration and invasion under normoxia and hypoxia. In addition, levels of phosphorylated FAK (Tyr 397), an important kinase mediating cell adhesion, were reduced in U87-shPLOD2 and U251-shPLOD2 cells, particularly under hypoxic conditions. Finally, orthotopic U251-shPLOD2 xenografts were circumscribed rather than locally invasive. In conclusion, the results indicated that *PLOD2* was a gene of clinical relevance with implications in glioblastoma invasion and treatment strategies.

## INTRODUCTION

Glioblastoma multiforme (WHO grade IV) is the most common and fatal primary malignant tumor in the brain. Despite advances in surgical and medical therapy, the life expectancy of patients with glioblastoma averages a mere 14 months following diagnosis [1]. The diffuse infiltration of glioblastoma into the surrounding brain parenchyma makes complete resection of the tumor impossible and is believed to be the major factor responsible for the resistance of glioblastoma to treatment [1]. Therefore, investigation of molecular mechanisms driving tumor invasion is essential for the development of a curative therapy of the disease.

To fully understand glioblastoma invasion requires some understanding of unique and complex tumor microenvironment as well as tumor cells. A hallmark characteristic of the glioblastoma microenvironment is hypoxia, which is associated with tumor growth, progression and resistance to conventional cancer therapies. Hypoxia prevents the degradation of hypoxia-inducible factor (HIF)-1/2α, which dimerizes with the constitutively active subunit, HIF-1β, to recruit transcriptional co-activators and initiate gene transcription [2]. How hypoxia regulates specific proteins involved in invasion might therefore help in the development of new therapies.

A second critical change in the tumor microenvironment is in the extracellular matrix (ECM). During tumor progression, ECM stiffness increases, as a result of increased collagen deposition and cross-linking, and it has been shown to enhance cancer cell invasion and metastasis [3, 4]. Increased ECM stiffness is commonly reported in solid cancers, including glioma. Collagen, one of the major components in ECM, has been associated with the development of tumor vessels and tumor adhesion in glioblastoma [5, 6]. The formation of collagen cross-links is initiated by procollagen-lysine 2-oxoglutarate 5-dioxygenase 2 (PLOD2), which specifically hydroxylates lysine residues of the collagen telopeptide area [7]. Interestingly, the role of PLOD2 has been reported in fibrous diseases [7, 8], but not in glioma. Through histology-based expression profiling, PLOD2 has been identified as a novel prognostic marker in glioblastoma [9]. Therefore, illuminating the expression and function of PLOD2 in glioblastoma may lead to the development of additional therapeutic strategies.

Here, we mined glioma microarray datasets to examine the expression profile of *PLOD2* in the development of human glioma. We subsequently investigated the role of PLOD2 in migration and invasion of glioblastoma in *in vitro* and *in vivo* systems. Our findings indicate that overexpression of PLOD2 is induced by hypoxia and promotes invasion and migration of glioblastoma cells. These results highlight PLOD2 as a potential new target for the inhibition of glioblastoma invasion.

## RESULTS

### *PLOD2* expression is positively correlated with tumor grade and inversely associated with glioblastoma patient prognosis

**Figure 1:**
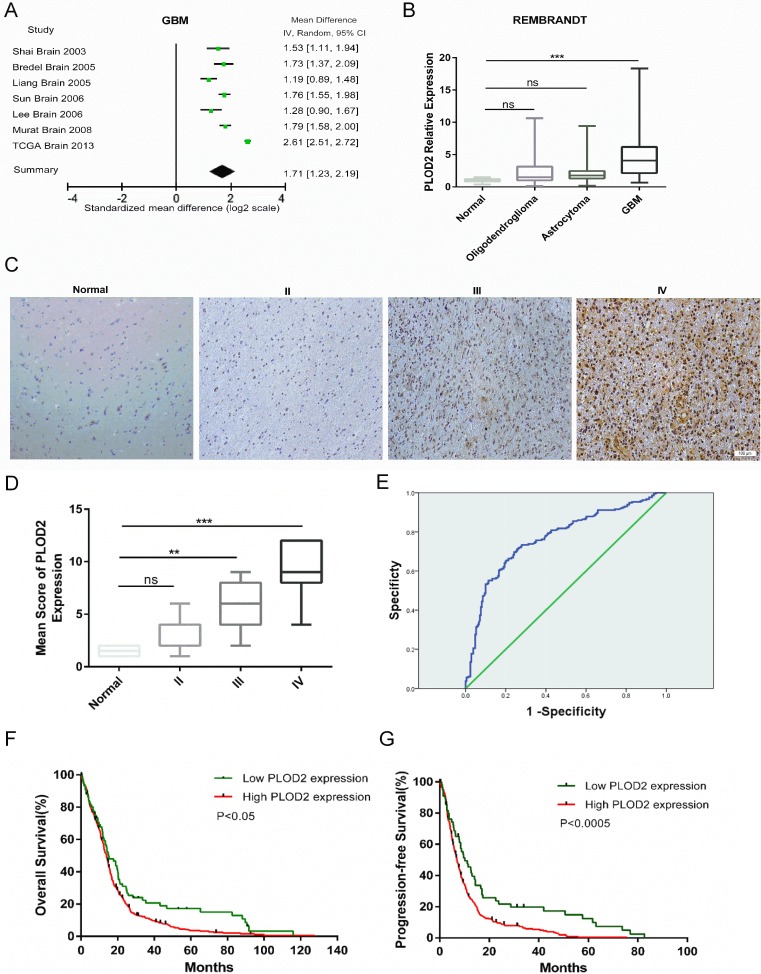
*PLOD2* is overexpressed in glioblastoma and correlates with poor prognosis (**A**) Forest plot of *PLOD2* expression levels in glioblastoma (*n* = 861) versus non-neoplastic brain tissue samples from the publicly available Oncomine datasets. The x-axis is the standardized mean difference between glioblastoma and normal *PLOD2* expression based on a log2 scale. (**B**) Expression of *PLOD2* mRNA in human gliomas of all grades (*n* = 178) from cases in the publicly available REMBRANDT database. (**C**) Representative images of immunohistochemical staining for PLOD2 in sections (4 μm) from primary human tumor samples (Scale bars, 100 μm). (**D**) Comparison of *PLOD2* mRNA levels among human gliomas based on tumor grade. Mean ± SD, **P < 0.01; ***P < 0.001; ns, not significant, versus normal samples. (**E**) ROC curve showing sensitivity of *PLOD2* as a marker to distinguish non-glioblastoma and glioblastoma patients. (**F** and **G**) Kaplan-Meier analyses for overall survival (OS, *n* = 522) and progression-free survival (PFS, *n* = 386) for glioblastoma patients in TCGA cohorts based on *PLOD2* expression levels (OS: *P* < 0.05; PFS: *P* < 0.0005, by the log-rank test).

To determine whether *PLOD2* was differentially expressed between glioblastoma and normal tissues, we broadly examined microarray data from patient samples within the Oncomine database. A meta-analysis of 7 independent glioblastoma datasets, consisting of 861 human glioblastoma samples, revealed that *PLOD2* was significantly and consistently overexpressed in glioblastoma across all datasets (Figure 1A, Mean Difference IV Random:1.71; CI: 1.23–2.19; *P* < 0.001). Next, we analyzed expression data from the REMBRANDT database for *PLOD2* based on tumor grade (*n* = 178). *PLOD2* mRNA expression was found to be significantly increased (~ 3-fold; *P* < 0.0001) in glioblastoma samples compared to normal brain and lower grade gliomas (Figure 1B).

Immunohistochemistry demonstrated that increasing PLOD2 protein levels were associated with increasing tumor grade. PLOD2 was highly expressed in grade III astrocytomas and to a greater degree in glioblastoma, whereas staining was absent or weak and appeared in fewer cells in non-neoplastic brain tissue samples and grade II astrocytomas (Figure 1C). The expression pattern of PLOD2 was dependent on tumor grade, with the exception that no significant difference was observed between non-neoplastic brain tissue samples and grade II astrocytoma (Figure 1D).

Based on these results, we used the data from TCGA and REMBRANDT databases to determine whether *PLOD2* could be used as a biomarker to distinguish between non-glioblastoma and glioblastoma patients. The area under the receiver operating characteristic (ROC) curve was 0.785 (CI, 0.785–0.828), indicating that *PLOD2* might be effective as a diagnostic marker to distinguish glioblastoma from lower grade gliomas (Figure 1E). Furthermore, the Kaplan-Meier estimates based on the TCGA dataset showed significant differences in overall survival (OS) and progression free survival (PFS) between glioblastoma patients with low *PLOD2* expression and those with high expression (Figure 1F, *P* < 0.05; Figure 1G, *P* < 0.0005, by the log-rank test). The median OS among patients with low *PLOD2* expression was 14.95 months (95% CI, 9.484–20.052) as compared to 13.93 months (95% CI, 12.884–15.020) among those with high expression. The median PFS for glioblastoma patients with low and high *PLOD2* expression was 10.22 months (95% CI, 6.03–14.41) and 7 months (95% CI, 6.141–7.859), respectively.

### Hypoxia induces PLOD2 expression through HIF-1α in glioma cells

**Figure 2:**
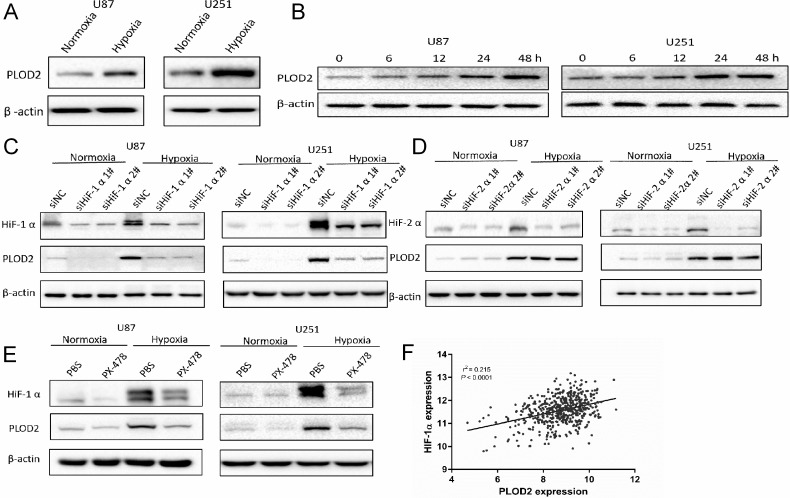
Hypoxia induces PLOD2 expression through HIF-1α in U87 and U251 glioma cells (**A**) Western blot for detection of PLOD2 levels in lysates prepared from U87 and U251 cells cultured under hypoxic conditions for 48 h. (**B**) Western blot for PLOD2 levels in U87 and U251 cultured 0, 6, 12, 24, 48 h under hypoxic conditions. (**C**) Western blot for PLOD2 and HIF-1α levels in U87 and U251 cells transfected with negative control siRNA (siNC) and HIF-1α siRNA (siHIF-1α#1-2; 100 nmol/L) and cultured under hypoxic conditions for 48 h. (**D**) Western blot for PLOD2 and HIF-2α levels in U87 and U251 cells transfected with negative control siRNA (siNC) and HIF-2α siRNA (siHIF-2α#1-2; 100 nmol/L) and cultured under hypoxic conditions for 48 h. (**E**) Western blot for PLOD2 and HIF-1α levels in U87 and U251 cells pre-treated with PX-478 (10 μM) and cultured under normoxic and hypoxic conditions for 48 h. β-actin was used as a loading control. (**F**) Correlation between *PLOD2* and *HIF-1α* mRNA levels as determined from the publicly available TCGA data (*n* = 528).

*PLOD2* mRNA has been reported to be induced by hypoxia in mouse fibroblasts [10]. To determine whether hypoxia induced expression of PLOD2 in human glioma cells, lysates prepared from cells incubated under hypoxic conditions for different time points (0 to 48 h) were examined by Western blot. PLOD2 protein levels increased in both U87 and U251 cells ~2 to 3-fold in a time dependent manner under hypoxia, compared to cells cultured under normoxic conditions (Figure 2A and 2B).

To investigate whether HIF-1α or HIF-2α were transcriptional regulators of *PLOD2* under hypoxia, siRNA knockdown experiments were performed. PLOD2 protein levels in U87 and U251 cells transfected with siHIF-1α were reduced under hypoxia as determined by Western blot (Figure 2C). However, no changes in PLOD2 expression were observed under hypoxia with transfection of siHIF-2α (Figure 2D). The role of HIF-1α in the regulation of PLOD2 was further investigated through exposure of glioma cells to the HIF-1α inhibitor PX-478. PX-478 is a novel agent that suppresses constitutive and hypoxia-induced HIF-1α by selectively inhibiting translation of the protein [11]. Inhibition of HIF-1α by PX-478 also led to reduced levels of PLOD2 protein in U87 and U251 cells under hypoxia (Figure 2E). Furthermore, *PLOD2* mRNA expression level was closely correlated to *HIF-1α* mRNA expression level (r^2^ = 0.215, *P* < 0.0001) based on the publicly available TCGA data (*n* = 528) (Figure 2F). Taken together, these results indicated that PLOD2 expression induced under hypoxia in glioma cells was mediated by HIF-1α.

### Decreased PLOD2 protein inhibits migration and invasion of glioma cells

**Figure 3:**
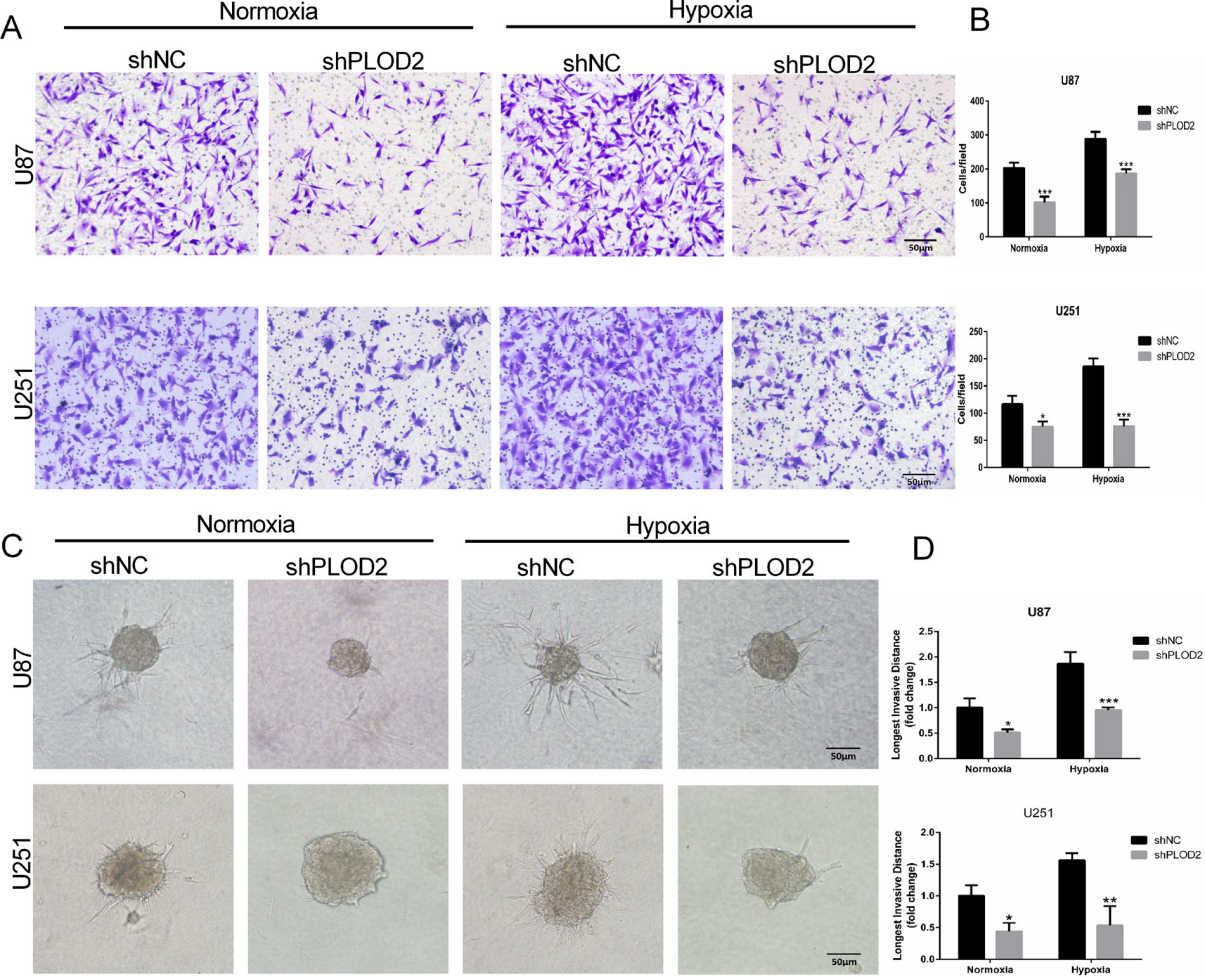
Decreased PLOD2 expression in U87 and U251 cells inhibits migration and invasion (**A**) Representative images from Transwell migration assays for U87-shNC and U87-shPLOD2 or U251-shNC and U251-shPLOD2 cells under normoxic or hypoxic conditions, as indicated, after 12 h (Scale bars, 50 μm). (**B**) Bar graphs representing quantification of the number of migrated cells per field for cell types indicated. (**C**) Representative images of 3D spheroid invasion assays for U87-shNC and U87-shPLOD2 or U251-shNC and U251-shPLOD2 under normoxic or hypoxic conditions for 24 h (Scale bars, 50 μm). (**D**) Bar graphs representing the longest invasion distance (fold change) for the cell type indicated. Data are presented as the mean ± SD (3 individual experiments). *P < 0.05; **P < 0.01; ***P < 0.001; versus shNC groups under the same condition; Student's *t* test.

The role of PLOD2 in glioma migration and invasion was examined *in vitro*. Although hypoxia promoted migration of controls, loss of PLOD2 inhibited migration in shPLOD2 expressing clones (~ 0.3-fold, *P* < 0.001, Figure 3A and 3B). In 3D culture, U87 and U251 cells grew into more structurally well-organized spheres, and formed the longest invasive projections surrounding the spheres under hypoxic conditions. Knockdown of PLOD2 inhibited the distance cells invaded into the Matrigel under both conditions (Figure 3C and 3D). These results demonstrated that PLOD2 function contributed to glioma cell migration and invasion *in vitro*, especially under hypoxia.

### PLOD2 promotes migration and invasion through FAK signaling in glioma

**Figure 4:**
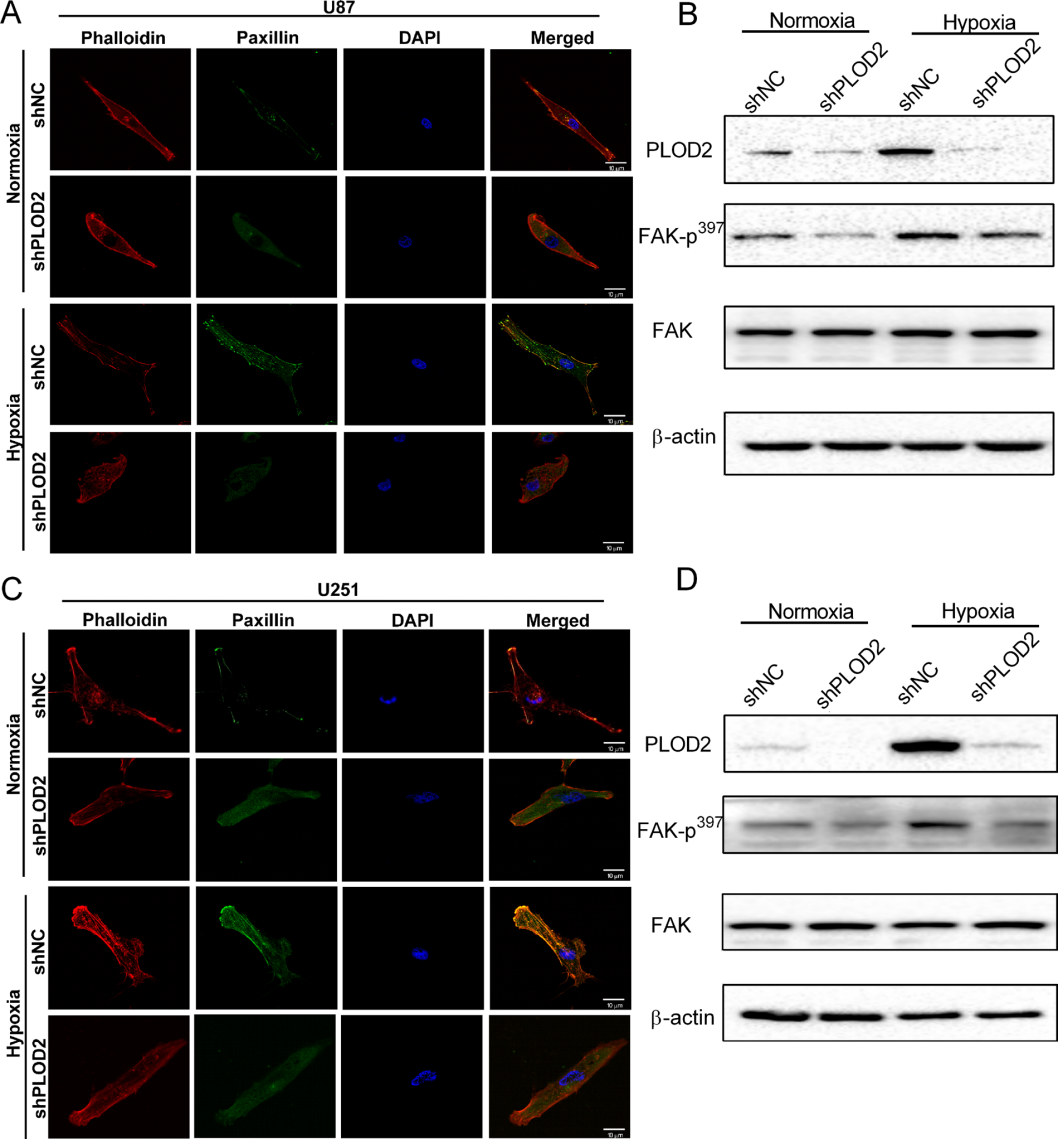
PLOD2 mediates actin cytoskeleton remodeling through FAK (**A**) Representative confocal images of U87-shNC and U87-shPLOD2 cells under normoxic and hypoxic conditions (Scale bars, 10 μm). Blue: DAPI (nuclei); red: phalloidin (polymerized actin/stress fiber); green: paxillin (focal adhesion). (**B**) Western blot for the detection of FAK-p^397^ levels in U87-shNC control and U87-shPLOD2 cells under normoxic and hypoxic conditions. (**C**) Representative confocal images of U251-shNC and U251-shPLOD2 cells under normoxic and hypoxic conditions (Scale bars, 10 μm). (**D**) Western blot for the detection of FAK-p^397^ levels in U251-shNC control and U251-shPLOD2 cells under normoxic and hypoxic conditions.

We next sought to identify molecular pathways that might mediate the involvement of PLOD2 in tumor cell migration and invasion. One way to evaluate migration and invasion is through expression of MMPs [12]. However, knockdown of PLOD2 did not lead to alterations in MMP-2 and MMP-9 protein levels under normoxic or hypoxic conditions in U87 or U251 cells (Supplementary Figure 1). A second approach to evaluate migration and invasion is through characterization of cell morphology and cytoskeletal status [13]. Cytoskeletal organization and adhesion are controlled by the complex coordination of actin cytoskeletal rearrangement and changes in focal adhesions [14]. These are cell characteristics that can be easily visualized under confocal microscopy by staining for paxillin with antibodies and actin fibers with phalloidin. Paxillin is a protein that is present in focal adhesions and is believed to play a role in mediating intracellular signaling in this process [15], as it co-localizes with actin fibers in activated focal adhesion plaques. Hypoxia significantly increased focal adhesions in both U87-shNC and U251-shNC, as determined by increased co-localization of paxillin (paxillin; green fluorescence) with actin fibers (phalloidin; red fluorescence) in focal adhesion plaques, compared to cells under normoxic conditions (Figure 4). Knockdown of PLOD2 however led to decreased formation of focal adhesion plaques and greater disorganization of actin stress fibers under both normoxic and hypoxic conditions (Figure 4A and 4C).

**Figure 5:**
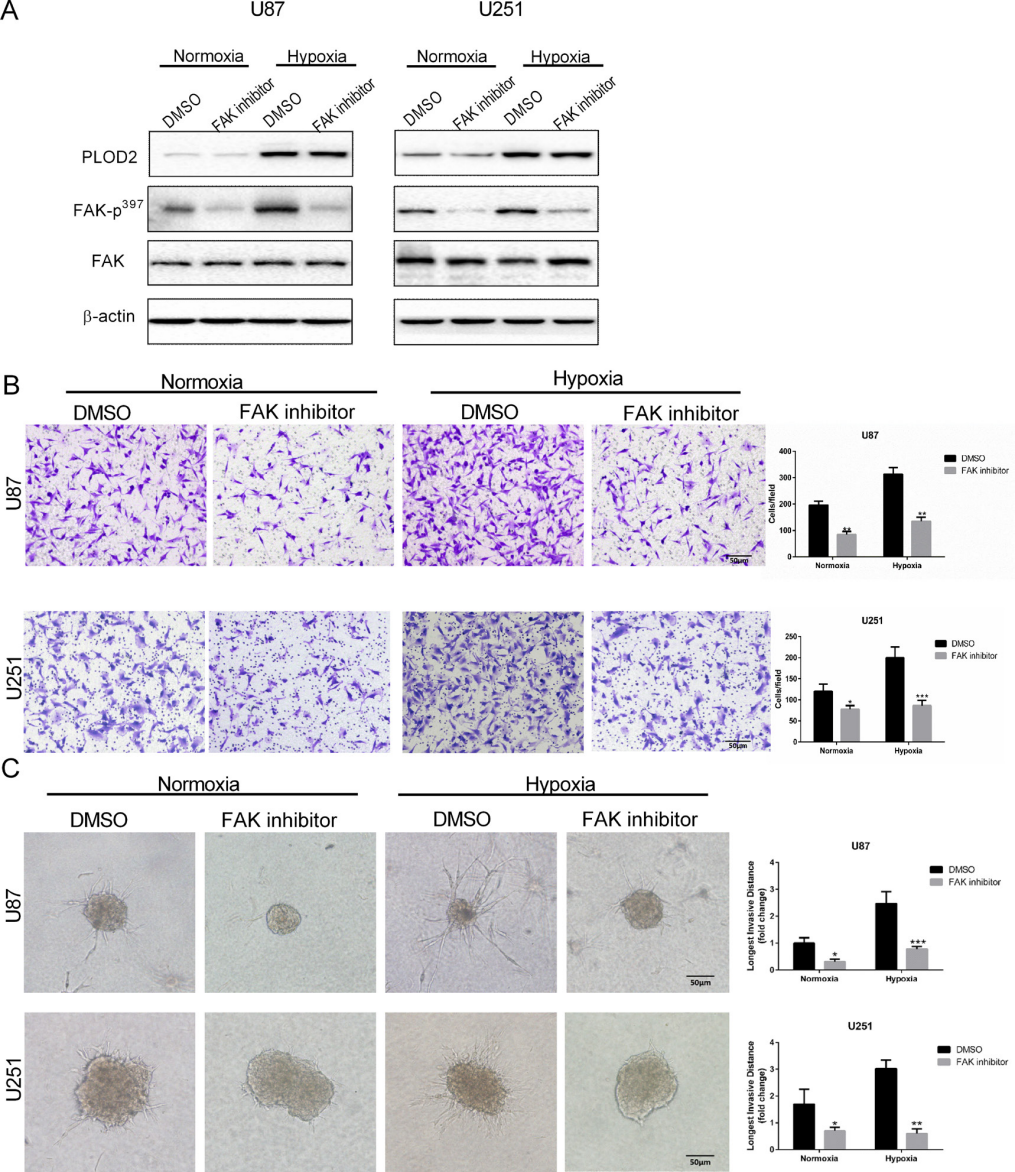
Treatment with FAK inhibitor attenuates U87 and U251 cell migration and invasion (**A**) Western blot for the detection of FAK-p^397^ levels in total protein lysates (20 μg) from U87 and U251 cells treated with FAK inhibitor TAE226 (5 μM) for 12 h under normoxic and hypoxic conditions. (**B**) Representative images and quantification (cell number/field) of transwell migration assays for parental U87 and U251 cells treated with TAE226 (5 μM) under normoxic and hypoxic conditions (Scale bars, 50 μm). (**C**) Representative images and quantification of 3D invasion assays (longest invasive distance) for U87 and U251 cells treated with TAE226 (5 μM) under normoxic and hypoxic conditions (Scale bars, 50 μm). Results are presented as the mean ± SD (*n* = 3). *P < 0.05; **P < 0.01; ***P < 0.001, versus U87-shNC or U251-shNC controls under the same conditions; Student's *t* test.

FAK plays a key role in modulating cell morphology and migration and is activated by cell adhesion or contact with ECM proteins. Phosphorylation of FAK was therefore investigated as a candidate signaling pathway mediating PLOD2 activity. Levels of phosphorylated FAK (Tyr 397) on Western blot were reduced after knockdown of PLOD2 in U87 and U251 cells, particularly under hypoxic conditions. These results indicated that PLOD2 might enhance the motility of cancer cells through FAK signaling (Figure 4B and 4D). In addition, glioma cells were exposed to the FAK inhibitor TAE226 under normoxia and hypoxia. Western blot confirmed that TAE226 inhibited phosphorylation of FAK (Tyr 397) under both normoxic and hypoxic conditions (Figure 5A). Furthermore, migration and 3D invasion of parental U87 and U251 were inhibited by treatment with TAE226 (Figure 5B and 5C). These data indicated that PLOD2 promoted glioma migration and invasion through FAK signaling.

### PLOD2 enhances glioma cell invasion *in vivo*

**Figure 6:**
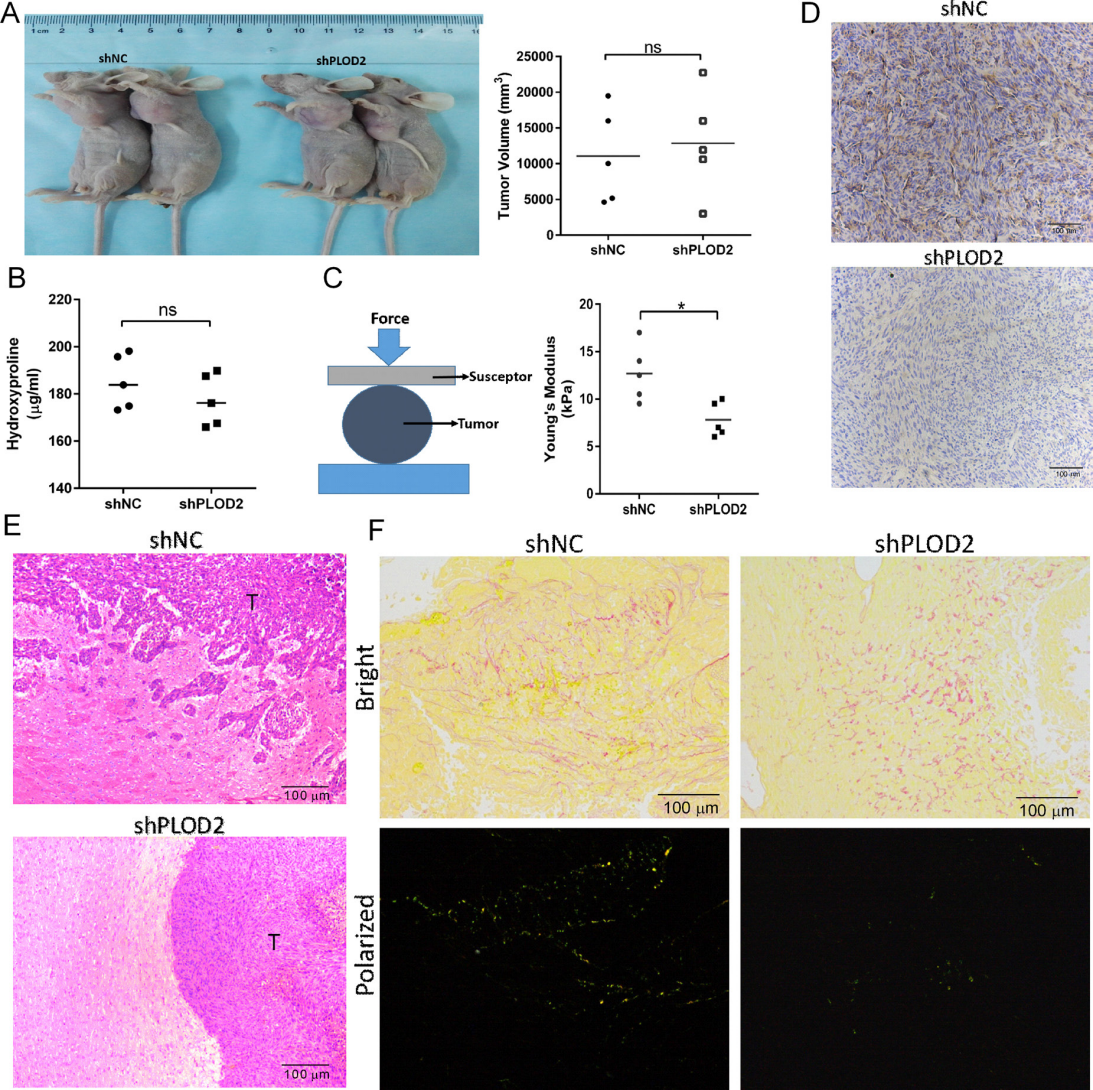
Knockdown of PLOD2 inhibits glioma cell invasion *in vivo* (**A**) Nude mice bearing subcutaneous xenografts of injected U251-shPLOD2 or U251-shNC cells (U251-shPLOD2, *n* = 5; U251-shNC, *n* = 5). Subcutaneous xenograft tumor volume (mm^3^) was measured on day 43 with a caliper. (**B**) Concentration of hydroxyproline (μg/mL) in U251-shNC or U251-shPLOD2 subcutaneous xenografts as determined by ELISA. (**C**) Stiffness of U251-shNC or U251-shPLOD2 subcutaneous xenografts measured with the compression test. Measurements were made on 3 randomly chosen sites in each tumor. ns, not significant, Student's *t* test. (**D**) PLOD2 immunohistochemical staining of tissue sections from U251-shNC or U251-shPLOD2 orthotopic xenografts (Scale bars, 100 μm). (**E**) Hematoxylin and eosin staining of sections at tumor margins in intracranial U251-shNC or U251-shPLOD2 xenografts (Scale bars, 100 μm). (**F**) Picrosirius red staining of tumor sections from U251-shNC or U251-shPLOD2 intracranial xenografts captured under bright and polarized light (Scale bars, 100 μm).

In order to assess the function of PLOD2 *in vivo*, we generated subcutaneous and orthotopic xenografts and characterized them based on various parameters. First, tumor volume of subcutaneously inoculated U251-shNC and U251-shPLOD2 cells was similar among groups at 4 weeks after implantation (Figure 6A). Second, hydroxylproline levels, which were used as an indicator of collagen content, were not significantly different between groups (Figure 6B). These *in vivo* results were consistent with those from *in vitro* demonstrating that overall collagen I levels in shPLOD2 cells did not differ from control shNC clones under normoxic or hypoxic conditions (Supplementary Figure 2). Third, stiffness measurements made with compression and indentation tests demonstrated that knockdown of PLOD2 reduced tumor stiffness (*P* < 0.05, Figure 6C). Fourth, pathological analysis of orthotopic xenografts revealed that U251-shNC cells generated tumors with irregular, locally invasive borders, whereas U251-shPLOD2 tumors were more circumscribed (Figure 6D and 6E). These results indicated that PLOD2 promoted glioma invasion. Finally, Picrosirius red staining, which is used to highlight collagen crosslinking, confirmed that PLOD2 was active in tumors. Collagen in U251-shNC tumors (control) was more cross-linked than in U251-shPLOD2 tumors (Figure 7F). Taken together, these data demonstrated that PLOD2 promoted tissue invasion of human glioma cells.

## DISCUSSION

The invasive phenotype of glioblastoma is a major cause for poor prognosis in glioma patients [16]. Invasion is a multi-step and complex process involving the interactions of cancer cells with the tumor microenvironment, including ECM [17]. In previous studies, PLOD2 expression was strongly linked to the progression of these diseases, most likely by increasing cross-linking of the collagen molecules that surround these tumors [18–20]. Here, we examined the expression and function of PLOD2 in glioblastoma with an emphasis on its possible role in invasion.

Several studies have observed that an enriched collagen I tumor microenvironment promoted metastasis by activating the β1-integrin-signaling pathway in breast cancer cells [21]. PLOD2 mediated collagen cross-linking has also been shown to further promote the development of a fibrotic microenvironment that enhanced breast cancer cell survival and facilitated metastasis to the lung and lymph nodes [22]. In the present study, functional studies *in vitro* demonstrated that depletion of PLOD2 did not lead to a reduction in collagen I levels under normoxic or hypoxic conditions (data not shown). However, after knockdown of PLOD2, cell migration and invasion were inhibited, particularly under hypoxic conditions. Such results indicated that PLOD2 may be an important driver of glioblastoma invasion.

A key process in cell invasion is the ability of a cell to stably adhere to ECM [23]. The process is regulated by one key protein within cells: FAK [24]. Previous studies have suggested that FAK critically promotes cell migration and invasion in several cancers by upregulating focal adhesion complex formation and matrix metalloproteinase expression [25]. It is known that tumors respond to changes in ECM stiffness through integrin activation and subsequent phosphorylation of FAK, resulting in Rho/ROCK activation and remodeling of the actin cytoskeleton [26]. In addition, lysyl oxidase (LOX) is a secreted amine oxidase whose primary function is to drive collagen crosslinking [27]. LOX-mediated cross-linking has been proposed to promote breast cancer malignant progression by increasing ECM stiffness [3]. A direct relationship between LOX enzymatic activity and tissue stiffness has also been reported in colon cancer [28]. LOX-induced collagen cross-linking resulted in stiffening of the matrix around tumor cells. The stiffening of the microenvironment led to increased FAK/SRC phosphorylation and a more invasive phenotype. Therefore, we speculated that PLOD2, a critical factor that promotes collagen cross-linking and acts upstream of LOX, may also activate the FAK pathway, in order to promote cell invasion in glioma cells. Our data demonstrated that knockdown of PLOD2 led to a decrease in the formation of focal adhesion plaques and FAK phosphorylation (Tyr397). These results emphasize the importance of PLOD2 in promoting ECM remodeling, changes in the relative cell cytoskeletal network, and tumor cell invasion.

We also performed *in vivo* studies to assess the potential effects of PLOD2 on glioma invasion within the brain environment. The results indicated that PLOD2 promoted cross-linking of collagen fibers around tumor cells and increased tumor stiffness. Such functions of PLOD2 might underlie its ability to stimulate glioma cells to infiltrate the brain parenchyma.

In summary, we investigated the value of PLOD2 as a clinical biomarker and its functions in glioblastoma. Our results demonstrated that PLOD2 promoted invasion of U87 and U251 cells *in vitro* and *in vivo* possibly through remodeling of ECM stiffness. This study indicated that PLOD2 may represent a potential therapeutic target for the prevention of glioblastoma invasion. Drugs or specific inhibitors of PLOD2 may therefore block collagen fiber biogenesis and potentially improve survival rates in patients with glioblastoma.

## MATERIALS AND METHODS

### Ethics statement

All experiments performed with human samples were approved by the Ethics Committee of Qilu Hospital of Shandong University (Jinan, China), and the study was conducted in accordance with the Declaration of Helsinki and international guidelines. Written informed consent was obtained from all patients. All studies involving animals were approved by the Institutional Animal Care and Use Committee, Qilu Hospital, Shandong University.

### Statistical analysis of microarray data

Meta-analysis of *PLOD2* gene expression in human glioblastoma and normal tissues as well as related statistical analysis was conducted using the Oncomine gene expression database (www.oncomine.com, Compendia Biosciences; Ann Arbor, MI, USA). The data were downloaded from Oncomine (TCGA, Sun Brain 2006 [29], Shai Brain 2003 [30], Bredel Brain 2005 [31], Liang 2005 [32], Lee 2006 [33], Murat 2008 [34]), and analysis was performed as previously described [35]. A univariate classification model for predicting glioblastoma versus non-glioblastoma based on gene expression across the independent datasets was evaluated in receiver operating characteristic (ROC) curves, as previously described [36].

### Patients and samples

Tissue samples (*n* = 78) from patients diagnosed with glioma were obtained during surgical resection at the Department of Neurosurgery, Qilu Hospital of Shandong University. None of the patients had received preoperative adjuvant therapy. Glioma specimens were histologically examined and classified according to the WHO classification criteria by two experienced clinical pathologists. Pathological diagnoses were distributed among tumor grades as follows: grade II, *n* = 20; grade III, *n* = 20; and grade IV, *n* = 29). Non-neoplastic brain tissues (*n* = 9) were collected from patients undergoing internal decompression surgery following severe traumatic brain injury.

### Antibodies and reagents

Antibody to PLOD2 for immunohistochemistry and Western blot was purchased from Proteintech (Wuhan, China). HIF-1α (PX-478) and FAK inhibitors (TAE226; Selleckchem; Shanghai, China) were resolubilized in DMSO. Matrigel was purchased from BD Biosciences (San Diego, CA, USA). The primary human antibodies used in this study were the following: rabbit anti-HIF-1α, anti-HIF-2α, anti-FAK-p^397^, anti-FAK, Paxillin (Abcam; Cambridge, MA, USA); mouse anti-β-actin (Beyotime, China).

### Immunohistochemical staining

Sections (4 μm) were cut from paraffin-embedded samples and then processed for immunohistochemistry. Briefly, the sections were deparaffinized, rehydrated, boiled in 1 mM EDTA (pH 8.0) for antigen retrieval and blocked with goat serum. Sections were incubated with primary antibodies overnight at 4°C (PLOD2 at 1:100; Proteintech; Wuhan, China), rinsed and incubated with poly-HRP secondary antibodies for 30 min. The substrate used for visualization was 3′-diaminobenzidine, and nuclei were counterstained with hematoxylin. Images were captured using an Olympus IX81 microscope. Blinded review of the specimens was performed independently by two pathologists. The immunoreactivity of PLOD2 was semiquantitatively scored with a well-established system incorporating both the percentage of positive tumor cells and the intensity of staining (5 fields of view per section). In short, a mean percentage of positive cells was determined among 5 fields at 200×, and the case was assigned to one of five categories: < 5% (0), 5–25% (1), 25–50% (2), 50–75% (3), and ≥ 75% (4). The mean intensity of staining was also determined in 5 fields and scored as: weak (1), moderate (2), and strong (3).

### Cell culture and cell treatment

Human glioma cell lines U87 and U251 were obtained from the Cell Bank of Type Culture Collection of Chinese Academy of Science (CBTCCCAS; Shanghai, China) and maintained in Dulbecco's modified Eagle's medium containing 10% FBS and antibiotics at 37°C in a humidified 5% CO_2_ incubator. The cell lines had been recently authenticated based on DNA fingerprinting, isozyme detection and cross species checks. For hypoxic treatment, cells at 50–60% confluence were incubated in a hypoxia chamber (5% CO_2_, 94% N_2_ and 1% O_2_).

### Transient transfection and lentiviral transduction

siRNAs against HIF-1α and HIF-2α were synthesized (GenePharma; Shanghai, China) with the following sequences: siHIF-1α-1: 5′-TACGTTGTGAGTGGTATTATT-3′ and siHIF-1α-2: 5′-CTGATGACCAGCAACTTGA-3′; siHIF-2α-1: 5′-CCCGGATAGACTTATTGCCAA-3′ and siHIF-2α-2:5′-TCACAGAACTGATTGGTTA-3′. For transfection, cells were plated at a density of 3 × 10^5^ cells/well in 6-well plates. When the cells were 70–80% confluent, the siRNA duplexes were transfected into cells using Lipofectamine 2000 (Thermo Fisher Scientific; Waltham, MA, USA) for 48 h. The cells were analyzed using Western blot analysis and immunofluorescence. Lentiviral vectors expressing human shRNA against *PLOD2* (shPLOD2; PIEL112078212, Shanghai GeneChem Company; Shanghai, China) or scrambled-control (shNC) were used to generate stable cell clones expressing shPLOD2 or a nonspecific shRNA as the control. Transfected clones were selected using 1 mg/mL of puromycin (Selleckchem, China). Western blot analysis was used to evaluate shRNA knockdown efficiency.

### Western blot analysis

Cell lysates containing total protein (20 μg) were electrophoresed on 10% SDS-PAGE and transferred onto a nitrocellulose membrane. Membranes were blocked in 5% skim milk blocking buffer for 1 h and incubated with primary antibodies overnight at 4°C. Membranes were rinsed and incubated with horseradish peroxidase-conjugated secondary antibody for 1 h. The protein bands were visualized using enhanced chemiluminescence (ECL; Thermo Fisher Scientific). Primary antibodies used were the following: HIF-1α, HIF-2α, FAK-p^397^, and FAK (Abcam; Cambridge, MA, USA); PLOD2 (Proteintech; Wuhan, China), and β-actin (Beyotime, China).

### Enzymes linked immunosorbent assay (ELISA)

Hydroxyproline content in tumor tissue was measured using the Hydroxyproline Assay Kit according to the manufacturer's instructions (Solarbio; Beijing, China).

### Cell migration assay

Assays were performed in 24-well transwell chambers (8.0 μm diameter pore). Glioma cells (5 × 10^4^) in FBS-free medium were seeded in the upper chamber. Medium containing 10% FBS was added to the lower chamber. After 12 h, cells that did not migrate were removed using cotton tips. Cells that migrated to the lower surface were fixed with methanol, stained with crystal violet. Five random views were used to count the cells under bright field microscopy (100× magnification).

### 3D invasion assay

Matrigel (40 μL; BD Biosciences; San Diego, CA, USA) was evenly spread in each well of a 24-well plate, and plates were placed in a cell culture incubator for 15–20 min to allow the Matrigel to solidify. Glioma cells (1 × 10^4^) were plated into each well with media containing 5% Matrigel, and the media was changed every 4 days. The formation of 3D spherical protruding structures between glioma cells were photographed at 2-day intervals for a total of 10 days. Six different random fields under bright field (200 ×) were chosen, and the longest distance of invasion was counted in each field with Imagine J software. Quantitation data are presented as the mean ± SD of results from 5 representative fields.

### Cytoskeleton staining assay

Glioma cells transfected with shNC and shPLOD2 were seeded onto glass slides. After 48 h, the cells were rinsed once with PBS, fixed with 4% paraformaldehyde in PBS for 15 min, permeabilized with 0.1% Triton X-100 in PBS for 30 min at room temperature, and blocked for 1 h with 3% BSA in PBS. Cells were stained with anti-Paxillin antibody (1:100; Abcam) overnight at 4°C. F-actin and nuclei were stained with phalloidin (Beyotime Biotechnology) and DAPI nuclear stain (Southern Biotech; Birmingham, AL, USA), respectively. Slides were viewed using Olympus confocal microscopy.

### *In vivo* experiments

U251 cells (5 × 10^6^) stably infected with either shNC or shPLOD2 lentivirus were subcutaneously inoculated into nude mice. After 6 weeks, the tumor formation status was recorded. For the intracranial glioma model, stably infected U251 cells (1 × 10^6^) were injected into the right striatum of 5-week-old nude mice following standard protocols. Animals were euthanized and tumors harvested for histologic analysis 4 weeks after implantation.

### Picrosirius red staining

Tumor sections were stained with 0.1% picrosirius red (Abcam) and counterstained with Weigert's hematoxylin. To visualize fibrillar collagen, stained sections were imaged with an Olympus IX51 fluorescence microscope fitted with an analyzer (U-ANT) and polarizer (UPOT).

### Stiffness measurement

Stiffness of tumors was measured as previously described [37]. Briefly, a cylindrical plane-ended indenter (3 mm) placed on the mouse skin surface was used to indent the exact region of soft tissue where tumor was located. The tissue was indented at a speed of 0.5 mm/s for three cycles of loading and unloading with a maximum indentation depth of 4 mm. The Young's modulus of the tissue was calculated based on an equation as described previously [37].

### Statistical analysis

All statistical analysis and experimental graphs were performed using SPSS version 13.0 (2004; Chicago, IL, USA) and GraphPad Prism 6 (GraphPad; La Jolla, CA, USA). Statistical comparisons among groups were made using the Student's *t* test and ANOVA. Data are shown as the mean ± SD, and all experiments were repeated independently at least three times. The Kaplan-Meier method was used to estimate the OS and PFS curves (log-rank test). *P* < 0.05 was considered statistically significant.

## SUPPLEMENTARY MATERIALS FIGURES


