# Correction: Procollagen-lysine 2-oxoglutarate 5-dioxygenase 2 promotes hypoxia-induced glioma migration and invasion

**DOI:** 10.18632/oncotarget.28003

**Published:** 2021-07-06

**Authors:** Yangyang Xu, Lin Zhang, Yuzhen Wei, Xin Zhang, Ran Xu, Mingzhi Han, Bing Huang, Anjing Chen, Wenjie Li, Qing Zhang, Gang Li, Jian Wang, Peng Zhao, Xingang Li

**Affiliations:** ^1^ Department of Neurosurgery, Qilu Hospital of Shandong University and Brain Science Research Institute, Shandong University, Jinan 250012, China; ^2^ Institute of Basic Medical Sciences, Qilu Hospital of Shandong University, Jinan 250012, China; ^3^ Department of Neurosurgery, Jining No.1 People’s Hospital, Jining 272011, China; ^4^ Department of Biomedicine, University of Bergen, 5009 Bergen, Norway

**This article has been corrected:** Due to errors during the assembly of Figure 5B, incorrect images were placed in row 1, panel 4 and row 2, panel 4. The corrected Figure 5B, produced using the original data, is shown below. The authors declare that these corrections do not change the results or conclusions of this paper.

Original article: Oncotarget. 2017; 8:23401–23413. 23401-23413. https://doi.org/10.18632/oncotarget.15581

**Figure 5:**
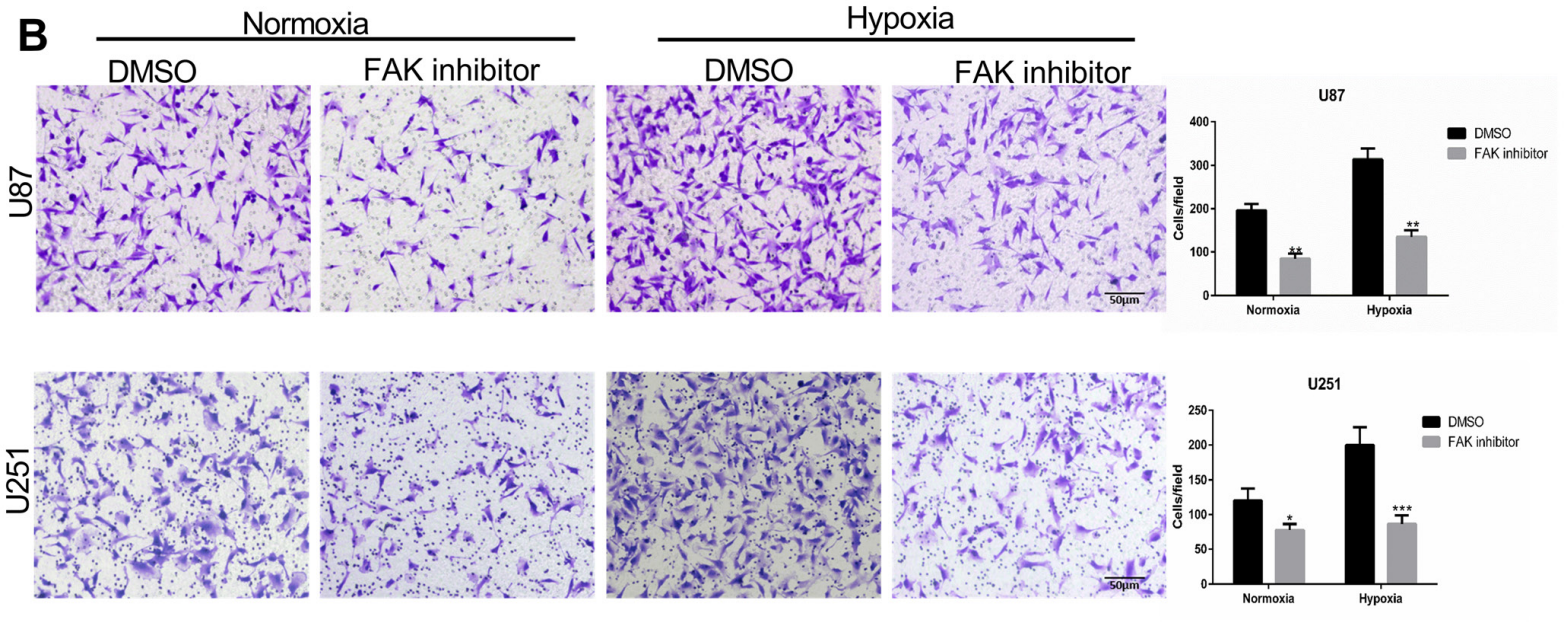
Treatment with FAK inhibitor attenuates U87 and U251 cell migration and invasion. (**B**) Representative images and quantification (cell number/field) of transwell migration assays for parental U87 and U251 cells treated with TAE226 (5 μM) under normoxic and hypoxic conditions (Scale bars, 50 μm).

