# Supplementary material for: Procollagen-lysine 2-oxoglutarate 5-dioxygenase 2 promotes hypoxia-induced glioma migration and invasion
Source: Oncotarget. 2017 Feb 21;8(14):23401–13. doi: 10.18632/oncotarget.15581 (PMC5410313; doi:10.18632/oncotarget.15581)
Supplement: Supplementary file 1 [file oncotarget-08-23401-s001.pdf]

Procollagen-lysine 2-oxoglutarate 5-dioxygenase 2 promotes hypoxia-induced glioma migration and invasion

Supplementary Materials

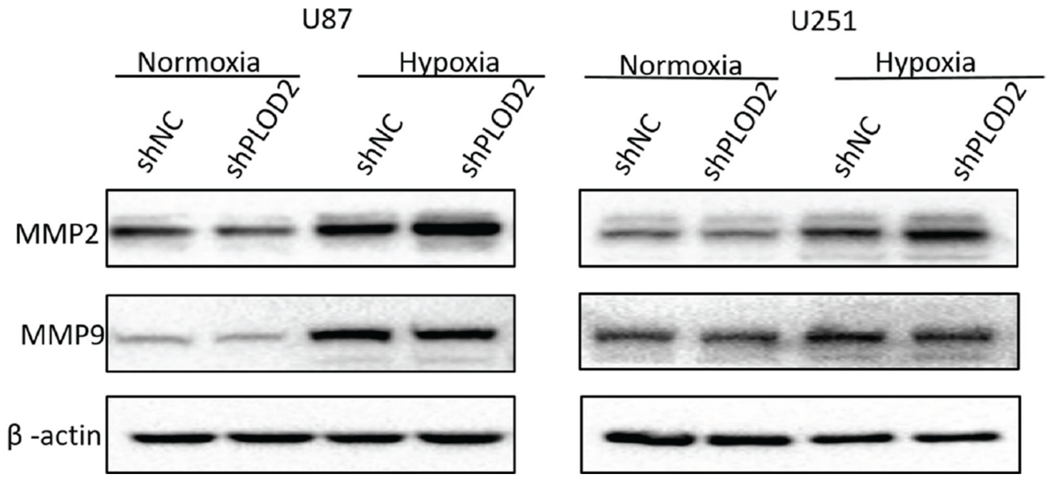

Supplementary Figure 1: PLOD2 does not regulate MMP2 and MMP9 protein levels. Western blot analysis of MMP2 and MMP9 in U87 and U251 cells infected with shPLOD2 or shNC lentivirus under normoxic or hypoxic conditions as indicated.

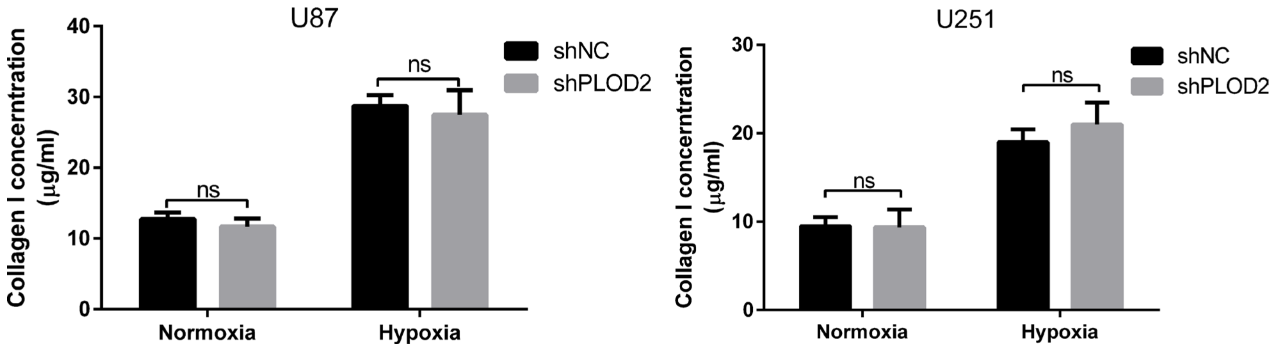

Supplementary Figure 2: Knockdown of PLOD2 does not lead to a decrease in collagen levels in U87 or U251 cells. ELISA used to measure collagen I concentrations (μg/mL) in conditioned media from U87-shNC and U87-shPLOD2 or U251-shNC and U251- shPLOD2 under normoxic or hypoxic conditions for 48 h. Data are presented as the mean ± SD (3 individual experiments); ns, not significant; versus shNC group under the indicated condition.
